# Regulation of UV-B-Induced Inflammatory Mediators by Activity-Dependent Neuroprotective Protein (ADNP)-Derived Peptide (NAP) in Corneal Epithelium

**DOI:** 10.3390/ijms24086895

**Published:** 2023-04-07

**Authors:** Grazia Maugeri, Agata Grazia D’Amico, Benedetta Magrì, Salvatore Giunta, Giuseppe Musumeci, Salvatore Saccone, Concetta Federico, Davide Scollo, Antonio Longo, Teresio Avitabile, Velia D’Agata

**Affiliations:** 1 Section of Anatomy, Histology and Movement Sciences, Department of Biomedical and Biotechnological Sciences, University of Catania, 95123 Catania, Italy; graziamaugeri@unict.it (G.M.); benedetta89@hotmail.it (B.M.); sgiunta@unict.it (S.G.); g.musumeci@unict.it (G.M.); 2 Section of System Biology, Department of Drug and Health Sciences, University of Catania, 95123 Catania, Italy; agata.damico@unict.it; 3 Section of Animal Biology, Department of Biological, Geological and Environmental Sciences, University of Catania, 95123 Catania, Italy; 4 Department of Ophthalmology, Eye Clinic, University of Catania, 95123 Catania, Italy; davidescollo@hotmail.com (D.S.); t.avitabile@unict.it (T.A.)

**Keywords:** cornea, ADNP, NAP, UV-B, IL-1β, NF-κB

## Abstract

The corneal epithelium, representing the outermost layer of the cornea, acts as a barrier to protect the eye against external insults such as ultraviolet B (UV-B) radiations. The inflammatory response induced by these adverse events can alter the corneal structure, leading to visual impairment. In a previous study, we demonstrated the positive effects of NAP, the active fragment of activity-dependent protein (ADNP), against oxidative stress induced by UV-B radiations. Here, we investigated its role to counteract the inflammatory event triggered by this insult contributing to the disruption of the corneal epithelial barrier. The results indicated that NAP treatment prevents UV-B-induced inflammatory processes by affecting IL-1β cytokine expression and NF-κB activation, as well as maintaining corneal epithelial barrier integrity. These findings may be useful for the future development of an NAP-based therapy for corneal disease.

## 1. Introduction

The cornea is a transparent tissue that acts as a barrier to protect the eye against external insults. It also provides two-thirds of the refractive power of the eye. The cornea is composed of five main layers: epithelium, Bowman’s layer, stroma, Descemet’s membrane, and endothelium. Besides epithelial cells, keratocytes, and endothelial cells, a heterogeneous population of antigen-presenting cells (APCs) reside in the cornea, including dendritic cells and macrophages [1]. In pathological conditions and during corneal wound healing, corneal epithelium and stroma are subjected to APC changes and infiltration of inflammatory cells, such as T lymphocytes [2,3,4]. The corneal epithelium is formed of approximately four layers of nonkeratinized stratified squamous epithelial cells with different morphologies. In fact, when proceeding from the inner to the outer layer, it is possible to identify the basal columnar, wing, and superficial squamous cells. Corneal epithelium protects the eye from the external environment. This function is made possible by the presence of tight junctions, formed by claudin, occludin, and membrane-associated proteins such as zonula occludens (ZO)-1, localized under a continuous pattern between epithelial cells [5].

The cornea is daily subjected to the solar ultraviolet B radiations (UV-B) whose wavelengths fall between 300 nm and 320 nm. It is well known that excessive exposure to UV-B can lead to photokeratitis and photo-ophthalmia, causing damage to the cornea [6]. At the cellular level, this insult evokes oxidative stress due to the localized production of reactive oxygen species (ROS) by corneal epithelial cells [7]. The oxidative stress induced by UV-B exposure triggers an inflammatory response mediated by resident and infiltrated cells. In response to various injurious stimuli, the corneal epithelial cells secrete into the extracellular space of different cytokines, such as interleukin (IL)-1β. This inflammatory factor damages the tight junction (TJ) proteins, such as ZO-1 and claudin, localized between adjacent epithelial cells [8,9,10]. Moreover, by acting through an autocrine and paracrine mechanism, IL-1β regulates the NF-κB signaling pathway. NF-κB is traditionally considered a central player in the inflammatory response. It activates various target genes, including pro-IL-1β, concurring to corneal epithelial barrier alterations [10,11,12]. Damage to the corneal epithelium can lead to a painful and inflammatory state that, in severe cases, can lead to vascularization and blindness if not adequately treated. Consequently, it is very important to identify new strategies and compounds to promote rapid re-epithelialization.

In recent years, several works have demonstrated the protective role of pituitary adenylate cyclase-activating polypeptide (PACAP) in the corneal epithelium and endothelium against insults of various kinds [13,14,15,16,17,18,19,20]. However, the short half-life of this peptide severely limits its therapeutic use [21]. PACAP plays its effects through the activation of G protein-coupled receptors [22]. Moreover, a subpicomolar concentration of PACAP stimulates the expression of activity-dependent protein (ADNP) [23]. ADNP is a neuroprotective protein of 123.56 kDa molecular weight that interacts with nuclear chromatin by regulating the transcription of genes involved in various biological events, such as embryogenesis, autophagy, and axonal transport [24,25]. ADNP is largely distributed throughout the body, including the eye [26]. Here, NAP (also known as davunetide), a small peptide of eight amino acids [23], exerts protective effects against retinal damage induced by different insults both in vivo and in vitro [27,28,29,30,31,32]. Moreover, in our recent paper, we showed the antioxidant role played by NAP in the cornea. In fact, NAP treatment decreased ROS generation by reducing apoptotic cell death and JNK signaling pathway activation induced by UV-B irradiation [33].

In the present study, we investigated the effect of NAP on the UV-B-induced inflammatory event and the subsequent epithelial corneal barrier damage. Our results showed that NAP counteracts the expression of IL-1β in corneal epithelial cells, affecting NF-κB activation and protecting the integrity of the corneal epithelial barrier. These data suggest that NAP or an analog thereof could be used in the treatment of corneal epithelial damage.

## 2. Results

### 2.1. NAP-Inhibited Inflammatory Cytokine IL-1β Expression in Corneal Epithelium Induced by UV-B

**Figure 1:**
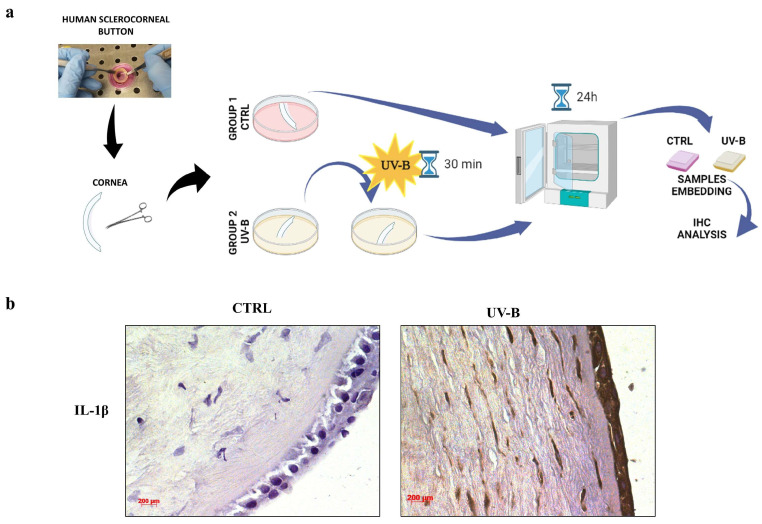
Effect of UV-B rays on IL-1β expression using an ex vivo human cornea model. (**a**) Image showing the experimental design for UV-B irradiation experiments. (**b**) Immunodetection of IL-1β in human cornea not (CTRL) or subjected to UV-B exposure (UV-B). Digital micrographs are representative results of fields taken in randomly selected slides and obtained using the Zeiss Axioplan light microscope (Carl Zeiss, Oberkochen, Germany) fitted with a digital camera (AxioCam MRc5; Carl Zeiss). Scale bar: 200 μm.

Excessive UV-B exposure causes an acute inflammatory event in the cornea. Since IL-1β is a pro-inflammatory cytokine playing a major role in inflammatory responses, we analyzed its expression after UV-B radiation damage in an ex vivo human cornea model (Figure 1a). As observed in Figure 1b, no IL-1β immunoreactivity was detected in human corneal epithelium control (CTRL). Instead, IL-1β staining was strongly increased in both the epithelium and in the stroma of human cornea exposed to 30 min of UV-B irradiation at a dose of 20 mJ/cm^2^.

**Figure 2:**
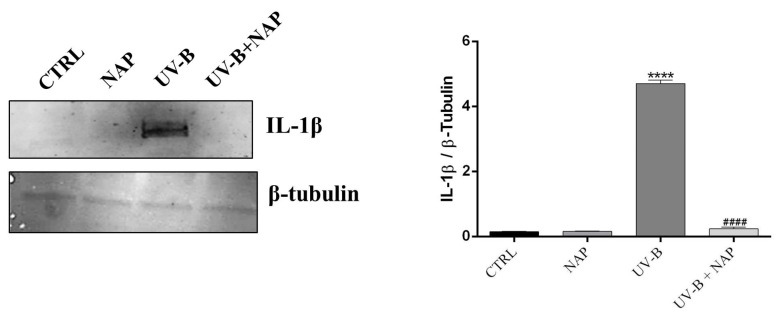
Effect of NAP treatment on IL-1β expression in UV-B-irradiated corneal epithelial cells. Representative immunoblots of IL-1β expression in SIRC cells cultured in either normal condition (CTRL) or with NAP (100 nM), or exposed to either UV-B alone (UV-B) or with NAP (UV-B+NAP). The bar graph shows a quantitative analysis of signals obtained by immunoblots resulting from three independent experiments. Data represent means ± SEM (**** *p* < 0.001 vs. CTRL; #### *p* < 0.001 vs. UV-B).

Next, the effect of UV-B to activate the inflammatory cascade was investigated in corneal epithelial cell cultures. The statens seruminstitut rabbit corneal (SIRC) epithelial cells were exposed to UV-B light for 30″ at a dose of 20 mJ/cm^2^. Immunoblot analysis revealed a significant increase in IL-1β expression 24 h after UV-B ray exposure as compared to control (**** *p* < 0.0001) (Figure 2). However, a remarkably reduced IL-1β expression was found in SIRC cells of the UV-B + NAP group when compared to the UV-B group (#### *p* < 0.0001) (Figure 2), suggesting that NAP exerts an anti-inflammatory role.

Therefore, we investigated whether NAP interfered with IL-1β secretion in SIRC cells exposed to UV-B radiations. As shown in Table 1, the secretion of IL-1β was significantly increased in corneal epithelial cells following the UV-B insult (**** *p* < 0.0001 vs. CTRL). The NAP treatment significantly reduced the cytokine release in the culture medium as demonstrated by the ELISA assay (### *p* < 0.001 vs. UV-B; Table 1).

**Figure 3:**
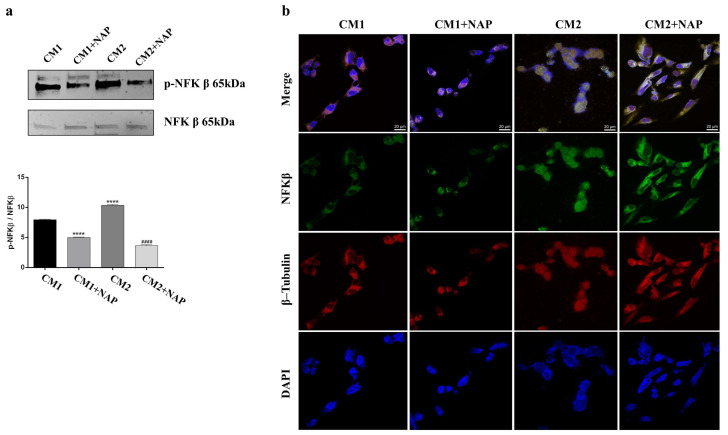
Effect of NAP treatment on the NF-κB activation in UV-B-irradiated corneal epithelial cells. (**a**) Representative immunoblots of p- NF-κB expression in SIRC cells cultured in normal condition (CM1) or with NAP (10 nM) (CM1 + NAP) or cultured with CM2 alone or with NAP (CM2+NAP). The bar graph shows a quantitative analysis of signals obtained by immunoblots resulting from three independent experiments. Data represent means ± SEM. (**** *p* < 0.0001 vs. CM1; #### *p* < 0.0001 vs. CM2). (**b**) Representative photomicrographs show NF-κB expression (red) and β-tubulin (green) in SIRC cells cultured in either normal condition (CM1) or with NAP (10 nM) (CM1 + NAP), or cultured with either CM2 alone or with NAP (CM2 + NAP). Photomicrographs are representative results of fields taken randomly from each slide. Scale bar, 20 µm.

The overexpression of pro-inflammatory cytokines such as IL-1β induces the activation of the NF-κB pathway, whose activation is required for the induction of several inflammatory genes [34]. In accord with our estimations, SIRC cells incubated with CM2, which were derived from corneal epithelial cells exposed to UV-B and containing 73 pg/mL IL-1β, showed a significantly increased phosphorylation of NF-κB as compared to control (**** *p* < 0.001 vs. CM1). Much less phosphorylation of NF-κB was found in CM2+NAP-treated SIRC cells than in cells cultured with CM2 (#### *p* < 0.0001 vs. CM2), suggesting the protective role of NAP treatment to counteract the inflammatory event. To establish NF-κB subcellular localization, immunofluorescence analysis was performed. As shown in Figure 3a, we found a predominant expression of NF-κB in the cytoplasm of CTRL (CM1) and CM1+NAP groups, whereas a preponderant nuclear NF-κB expression was detected in the CM2 group. Interestingly, the treatment with NAP in CM2-cultured cells seems to promote NF-κB translocation to the cytoplasm (Figure 3b).

### 2.2. NAP Counteracts Corneal Epithelial Barrier Dysfunction against UV-B-Induced Inflammation

**Figure 4:**
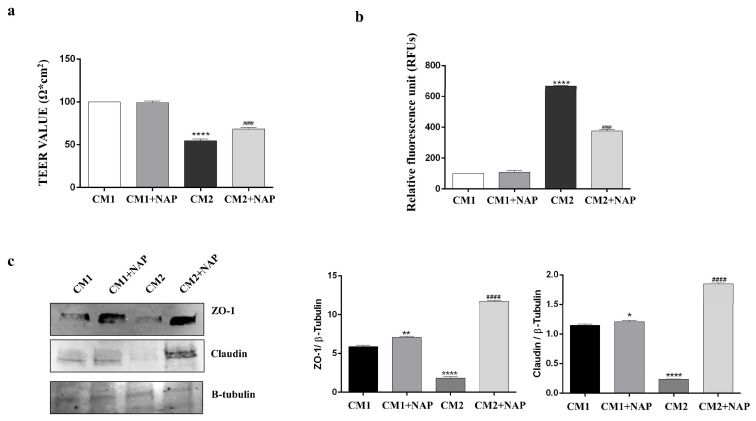
NAP attenuates the UV-B-induced barrier alterations of corneal epithelial cells. TEER (**a**) and permeability assays (**b**) were performed in SIRC cells grown on transwell clear permeable supports in either normal conditions (CM1) or with NAP (10 nM) (CM1+NAP), or cultured with either CM2 alone or with NAP (CM2 + NAP). Results are expressed as the mean ± SD. **** *p* < 0.0001 vs. CTRL; ### *p* < 0.001 vs. UV-B. (**c**) Expression of ZO-1 and claudin tight junction. The bar graphs show a quantitative analysis of signals obtained by immunoblots resulting from three independent experiments. Data represent means ± SD. * *p* < 0.05. ** *p* < 0.01 and **** *p* < 0.0001 vs. CM1; #### *p* < 0.001 vs. CM2.

UV-B irradiation elicits a wide range of events in the cornea, including an inflammatory response, which concurs with corneal epithelium damage. The over-release of cytokines and, in particular, of IL-1β damages the barrier of different types of epithelial cells, including retinal pigment epithelial cells and corneal epithelial cells [10,35]. To test the role of NAP on corneal epithelial barrier integrity impaired by UV-B-induced inflammatory insult, we performed trans-epithelial-electrical resistance (TEER) and permeability assays. SIRC cells were cultured on 12 well transwell membranes and incubated with 300 µL of CM1, CM1 + NAP, CM2 or CM2 + NAP for 24 h. As shown in Figure 4a, incubation of cells with CM2, derived from SIRC cells exposed to UV-B and containing 73 pg/mL IL-1β, significantly reduced TEER values as compared to vehicle-cultured SIRC cells (**** *p* < 0.0001 vs. CM1). Instead, a significant increase in TEER values was observed in SIRC cells grown with CM2+NAP containing 21 pg/mL IL-1β, compared to cells cultured with CM2 (#### *p* < 0.0001 vs. CM2). As expected, permeability values were inversely related to TEER results. Indeed, we found remarkable hyperpermeability in cells cultured with CM2 (Figure 4b; *** *p* <  0.001 vs. CM1). Instead, the permeability of the cellular layer was significantly reduced in cells grown with CM2+NAP (Figure 4b; #### *p* < 0.0001 vs. CM2).

To confirm the role of NAP to counteract corneal epithelial barrier dysfunction against UV-B-induced inflammation, we analyzed the expression of the tight junctions, ZO-1 and claudin, which play an important role in the formation and maintenance of the corneal epithelial barrier. Moreover, claudin is a TJ protein more highly expressed in SIRC cells as compared to others, such as occludin, as previously demonstrated by Olivieri et al. [36]. As shown in Figure 4c, the expression of ZO-1 and claudin was drastically decreased in cells cultured with the conditioned medium derived from SIRC cells exposed to UV-B (**** *p* < 0.0001 vs. CM1), whereas in cells cultured with CM2+NAP, the tight junctions’ levels significantly increased (#### *p* < 0.0001 vs. CM2).

## 3. Discussion

Alterations in the integrity of the corneal epithelial barrier, resulting from infection, keratopathy, dry eye, mechanical, chemical, or physical trauma, such as UV-B radiation, result in persistent corneal epithelial defects [37], which if not adequately treated can lead to corneal opacification and, in the end, loss of vision. In the worst cases, keratoplasty becomes necessary, so identifying molecules that exert a protective effect at the level of the corneal epithelium is a major challenge especially due to the limited availability of donor corneas.

Different works have shown that NAP, the active fragment derived from ADNP, exerts a protective role in different ocular diseases [27,28,29,30,32]. This is not a surprise, considering that the ADNP syndrome, also known as Helsmoortel–Van der Aa syndrome (HVAS) caused by ADNP mutations, is characterized by multiple clinical symptoms, including ophthalmic alterations. In our previous work, we demonstrated that exogenous treatment with NAP, representing the active fraction of ADNP, counteracts the generation of UV-B-induced ROS, reducing the activation of the JNK pathway and exerting an anti-apoptotic role [33]. Since UV-B exposure is a well-documented environmental stressor resulting not only in oxidative stress but also in inflammation, here, we investigated the role of NAP in counteracting the harmful effect of IL-1β induced by UV-B radiations.

IL-1β is one of the most important mediators in local acute inflammation. In our ex vivo human cornea model subjected to UV-B radiation damage, the expression of IL-1β was drastically increased as compared to the control (Figure 1), confirming the role of UV-B insult to activate the inflammatory cascade in the cornea. Furthermore, it has been reported that UV-B exposure possesses the ability to increase the expression of IL-1β in corneal cells [8]. As predicted, the expression and secretion of IL-1β were markedly elevated in corneal epithelial cells irradiated by UV-B rays (Figure 2, Table 1). In the current study, we demonstrated that NAP exerts an anti-inflammatory effect through the reduction in this cytokine expression. In fact, treatment with NAP suppressed the expression and release of IL-1β (Table 1 and Figure 2). These results confirm previous evidence demonstrating the anti-inflammatory effects of NAP treatment both in vitro and in vivo. In particular, NAP downregulated TNF-α, IL-16, and IL-12 expression in macrophages [38]. Moreover, the octapeptide reduced IL-1β and its related receptors in diabetic rat retina and exerted potent anti-inflammatory effects in both acute small intestinal and acute large intestinal inflammation [31,39,40,41].

It is well-known that proinflammatory cytokines, and in particular IL-1β, can concur with the alteration of the barrier function of the corneal epithelium [42]. To confirm this negative role on the corneal barrier, we cultured SIRC cells in conditioned media from cells exposed to UV-B containing the known concentration of IL-1β equal to 73 pg/mL (CM2). As shown in Figure 4, exposure to this inflammatory cytokine strongly affects corneal epithelial barrier integrity by reducing and increasing TEER value and permeability, respectively. This data is related to the reduction in the expression of TJ proteins, ZO-1 and claudin, as shown in cells cultured with CM2. The peptide treatment decreased the expression levels of IL-1β as well as its release (Figure 2 and Table 1). This reduction positively affected corneal epithelial barrier integrity as demonstrated by the reduction in permeability and increase in TEER values, as well as tight junction proteins expression observed when SIRC cells were cultured with CM2+NAP. The protective effects of NAP on the corneal epithelial barrier could be due not only to reducing IL-1β expression and secretion, but also to its interaction with microtubules/tubulin or microtubule-associated proteins. In fact, it is well known that NAP co-localizes with microtubules [43], and the latter participate to keep structure, function, and restoration of TJ [44].

**Figure 5:**
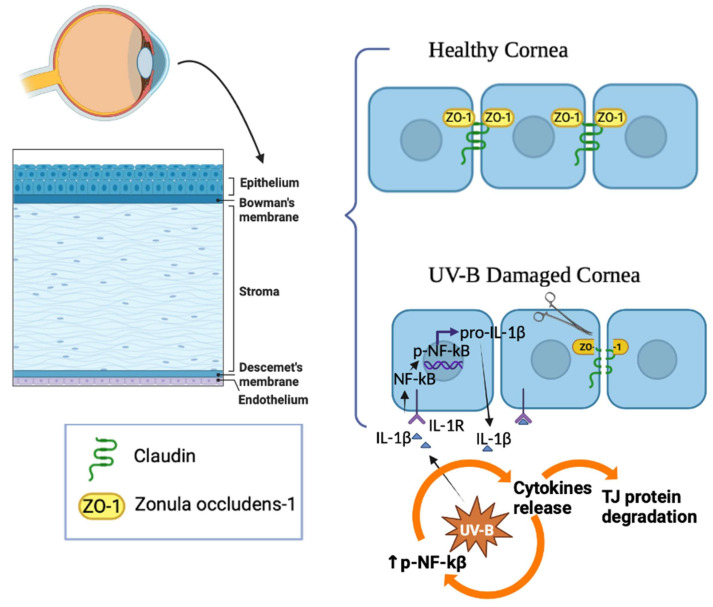
Schematic representation of the UV-B radiation-induced inflammatory event contributing to the disruption of the corneal epithelial barrier.

Our results also showed that IL-1β induced by UV-B radiations activates the NF-κB signaling pathway, which plays a central role in the vicious cycle of inflammation. In fact, UV-B radiation-induced overexpression and secretion of IL-1β that activate the NF-κB signaling pathway are in turn responsible for cytokine release (Figure 5). Moreover, it is well known that NF-κB is localized both in the cytoplasm and in the nucleus, and its intracellular distribution is dynamic. In particular, in response to the inflammatory process, NF-κB is translocated from the cytoplasm to the nucleus, where it regulates the transcription of NF-κB target genes [45]. A noteworthy point is that our data showed that the treatment with NAP downregulates NF-κB activation (Figure 3a) and promotes NF-κB translocation to the cytoplasm (Figure 3b). This study is thus only the starting point of a full characterization of the anti-inflammatory effect played by NAP. During inflammation, the excessive IL-1β release increases the inflammatory cascades and maturates APC cells, including macrophage. The latter are important producers of proinflammatory cytokines and chemokines, which in turn can act in an autocrine manner to promote other APC cells’ activation and maturation. Therefore, it will be important to investigate the role of NAP on these cells that could represent an important target in the management of corneal epithelial inflammation. One limitation of the present work is that the experiments were performed only on SIRC cells, whose monolayers do not perfectly reflect the in vivo characteristics of the corneal epithelium. Therefore, the promising results obtained needed validation in animal studies as well as clinical trials.

In conclusion, these data confirmed that UV-B-induced ocular inflammation is associated with IL-1β overproduction by corneal epithelial cells. This cytokine released in the extracellular space causes tight junction protein degradation between cells, resulting in increased cornel barrier permeability (Figure 5). Through an autocrine or paracrine action, IL-1β also stimulates the NF-κB pathway in corneal epithelial cells. NF-κB translocates to the participating nucleus to maintain this vicious cycle by stimulating IL-1β transcription (Figure 5). By counteracting the harmful effect of inflammatory players and maintaining the integrity of corneal epithelial tight junctions, NAP can be considered a protective agent in corneal epithelial disorders.

## 4. Materials and Methods

### 4.1. Ethics Statement

The study was performed in accordance with the tenets of the Declaration of Helsinki. The human sclerocorneal button stored in organ culture at 31 °C was supplied for penetrating keratoplasty by the Eye Bank (Fondazione Banca degli Occhi del Veneto; Venezia-Mestre, Italy), which obtained informed consent for all tissue samples held and cultured (Ethical approval number 99/2019/PO).

### 4.2. Human Cornea Damage Model

Human cornea deemed unsuitable for transplantation was obtained from Fondazione Banca degli Occhi del Veneto, Italy.

The human cornea was cut and divided into two groups: CTRL and UV-B, and maintained at 37 °C for a total of 24 h. Cornea samples of CTRL group were placed in a 5% CO_2_ incubator and culturing continued for 24 h. Cornea samples of UV-B group were subjected to ultraviolet irradiation at a dose of 20 mJ/cm^2^ by using an ultraviolet B (UV-B) lamp for 30 min and then placed in the incubator; culturing continued for 24 h.

### 4.3. Immunohistochemistry (IHC) Analysis

The expression and distribution of IL-1β in human cornea was evaluated through immunohistochemical analysis [46]. The sections were incubated overnight at 4 °C with IL-1β antibody (sc-1250; Santa Cruz Biotechnology; Dallas, TX, USA) at 1:50 work dilution in PBS and 1% BSA. Then, they were incubated with secondary antibodies conjugated to polymer-HRP (LSAB+ System-HRP, K0690, Dako, Denmark) and the immunoreaction was detected by incubating them for 3 min in a 3,30-diaminobenzidine solution (DAB substrate kit; SK-4100, Vector Laboratories, Burlingame, CA, USA). The samples were lightly counterstained with hematoxylin, mounted through vecta mount (Vector Laboratories) and observed with an Axioplan Zeiss light microscope (Carl Zeiss, Oberkochen, Germany), and finally photographed with a digital camera (AxioCam MRc5, Carl Zeiss, Oberkochen, Germany).

### 4.4. Cell Cultures

The statens seruminstitut rabbit corneal (SIRC) epithelial cells obtained from the American Type Culture Collection (ATCC) were grown in a minimum essential medium with Earle’s salts, l-glutamine, and non-essential amino acids supplemented (Eagle’s Minimum Essential Medium (ATCC^®^ 30-2003TM)) with 10% activated fetal bovine serum (FBS, 10108-165, GIBCO, Milan, Italy) and incubated at 37 °C in a humidified atmosphere of 5% CO_2_. Cells were cultured in a control medium (CTRL), or treated with 10 nM NAP (NAP) (New England Peptide, MA, USA), or subjected to ultraviolet irradiation at a dose of 20 mJ/cm^2^ by using an ultraviolet B (UV-B) lamp at 302 nm with a filter size of 21 cm × 26 cm (Uvitec, Cambridgeshire, UK) with (NAP+UV-B) or without 10 nM NAP (UV-B) for 24 h. The concentration of NAP is the same utilized in previous in vitro tests [33].

### 4.5. Immunofluorescence Analysis

To evaluate the cellular distribution of NF-κB and β-tubulin proteins, immunofluorescence analysis was performed on SIRC cells as previously described by Maugeri et al. [47]. The primary antibodies used for immunofluorescence analysis include: rabbit NF-κB (D14E12; Cell Signaling, Milan, Italy; 1:400), antibody, and mouse β-tubulin (sc-5274; Santa Cruz Biotechnology, Dallas, TX, USA; 1:500). Signals were revealed with Alexa Fluor, 488 goat anti-mouse (Waltham, MA, USA) and Alexa Fluor 594 goat anti-rabbit (Waltham, MA, USA), for 1.5 h at room temperature (shielded from light). DNA was counterstained with 4,6-diamidino-2-phenylindole (DAPI; cat. no 940110; Vector Laboratories, Burlingame, CA, USA). Immunolocalization was analyzed by confocal laser scanning microscopy (Zeiss LSM700, Oberkochen, Germany).

### 4.6. Western Blot Analysis

Western blot analysis was performed according to the procedures previously described [48]. About 20 μg of protein homogenate were diluted in 2× Laemmli buffer (Invitrogen, Waltham, MA, USA), heated at 70 °C for 10 min and then separated on a Biorad Criterion XT (Hercules, CA, USA) 4% to 15% bis-tris gel (Invitrogen, Waltham, MA, USA) by electrophoresis and then transferred to a nitrocellulose membrane (Invitrogen, Waltham, MA, USA). The primary antibodies used for the Western blot include: goat anti- IL-1β (sc-1250; Santa Cruz Biotechnology, Dallas, TX, USA; 1:200); rabbit anti-NF-κB (D14E12; Cell signaling, Milan, Italy, 1:1000); rabbit anti-p-NF-κB (S536; Cell signaling, Milan, Italy, 1:1000); and mouse anti-β-tubulin (sc-5274; Santa Cruz Biotechnology, Dallas, TX, USA; 1:200). The secondary antibody donkey anti-goat IRDye 680RD (926-68074; Li-Cor Biosciences; Lincoln, NE, USA); goat anti-rabbit IRDye 800CW (926-32211; Li-Cor Biosciences, Lincoln, NE, USA); and goat anti-mouse IRDye 680CW (926-68020D; Li-Cor Biosciences, Lincoln, NE, USA) were used at 1:20,000. Blots were scanned with an Odyssey infrared imaging system (Odyssey, Li-Cor Biosciences, Nebraska, NE, USA). Densitometric analyses of Western blot signals were performed at non-saturating exposures and analyzed using the ImageJ software (Version 1.53t). Values were normalized to β-tubulin, which served as a loading control.

### 4.7. Measurement of Trans-Epithelial-Electrical Resistance

The progress of epithelial barrier formation and polarization was monitored by measuring trans-epithelial-electrical resistance (TEER) using a Millicel-Electrical Resistance System (ERS2, Millipore, Epithelial Volt-Ohm Meter, Merck Millipore, Germany) as previously described [49]. TEER was recorded in SIRC cells grown on transwell supports. Measurements started after 15 min equilibration period at room temperature. Values are expressed as Ω × cm^2^.

### 4.8. Permeability Assay

Permeability assay on filter-cultured SIRC cells was performed by measuring the apical-to-basolateral movements of FITC-dextran solution with average mol wt 70,000 (Sigma–Aldrich, St. Louis, MI, USA) as previously described [50]. The FITC-dextran solution was added into the apical compartment. Sixty minutes later, the medium from the lower chamber was collected and the absorbance was measured through a microplate reader (Biorad 680, Milan, Italy) using 480 nm for excitation and 535 nm for emission.

### 4.9. Statistical Analysis

Data are represented as mean ± SEM. One-way analysis of variance was used to compare differences among groups, and statistical significance was assessed by the Tukey–Kramer post hoc test. The level of significance for all statistical tests was set at *p* ≤ 0.05.

## Figures and Tables

**Table 1:** IL-1β content in a conditioned medium from SIRC cells. IL-1β levels were detected in supernatants and expressed in pg/mL. Data resulting from three independent experiments are represented as means ± SEM. * *p* < 0.001 vs. CM1; ^#^ *p* < 0.001 vs. CM2.

SIRC Cell Line-Derived Conditioned Media	CTRL (CM1) Mean ± SEM	NAP Mean ± SEM	UV-B (CM2) Mean ± SEM	UV-B + NAP Mean ± SEM
IL-1β (pg/mL)	15.7 ± 3.65	15.2 ± 3.82	73 * ± 4.87	21 ^#^ ± 4.53

## Data Availability

The data presented in this study are available in article.
